# Thermochemical Measurements of Alkali Cation Association to Hexatantalate

**DOI:** 10.3390/molecules23102441

**Published:** 2018-09-24

**Authors:** Dylan J. Sures, G. P. Nagabhushana, Alexandra Navrotsky, May Nyman

**Affiliations:** 1 Department of Chemistry, Oregon State University, Corvallis, OR 97331-4003, USA; 2 Department of Chemistry, University of California, Davis, One Shields Avenue, Davis, CA 95616, USA; 3 Peter A. Rock Thermochemistry Laboratory and NEAT ORU, University of California Davis, Davis, CA 95616, USA; gpnbhushan@gmail.com (G.P.N.); anavrotsky@ucdavis.edu (A.N.)

**Keywords:** polyoxometalates, hexatantalate, tantalum, countercations, ion-pairing, calorimetry, solubility

## Abstract

Ion association is an important process in aqueous dissolution, precipitation, and crystallization of ionic inorganic, organic, and biological materials. Polyoxometalates (POMs) are good model compounds for understanding the complex relationships between lattice energy, ion-pairing in solution, and salt solubility. Here we perform calorimetric measurements to elucidate trends in cluster stability, lattice energy, and ion-pairing behavior studies of simple hexatantalate salts in neat water, parent hydroxide solutions, and molybdate melts, extending previous studies on the isostructural hexaniobates. High temperature calorimetry of alkali salts of hexatantalate reveals that the enthalpies of formation from oxides of the K, Rb, and Cs salts are more similar to each other than they are for their niobate analogues and that the tantalate cluster is energetically less stable than hexaniobate. Aqueous dissolution calorimetry reveals that the cesium salt of hexatantalate has a similar concentration dependence on its dissolution enthalpy to that of hexaniobate. However, unlike rubidium hexaniobate, rubidium hexatantalate also exhibits increased concentration dependence, indicating that hextantalate can undergo increased ion-pairing with alkali salts other than cesium, despite the dilute environments studied. Dissolution enthalpies of POM salts in the parent alkali hydroxides shows that protonation of clusters stabilizes lattices even more than the strongly associating heavy alkali cations do. Additionally, neither weak nor strong lattice ion associations necessarily correlates with respectively high or low aqueous solubility. These studies illuminate the importance of considering ion-pairing among the interrelated processes in the aqueous dissolution of ionic salts that can be extended to serving as a model of cation association to metal oxide surfaces.

## 1. Introduction

Aqueous ion behavior is driven by many fundamental and interrelated physical processes. The solubility of ionic salts in water is predictable to some degree by the “hardness” or “softness” of the component cations and anions, which arises from their degrees of hydration upon dissolution, electron count in the frontier molecular orbitals, overall charge, and charge density [1,2,3,4]. Typically, close interactions between cations and anions (ion-pairing) in solutions predicates precipitation, with larger hydration spheres from more charge-dense species (i.e., Li^+^) maintaining solubility, according to the Hoffmeister series [5]. This factor competes with lattice energy, which is a sum of energies of all the bonds present in the lattice. For simple monoatomic ions such as alkali halides, similarly sized cations and anions stabilize each other to the greatest degree, decreasing solubility. The relationship between lattice energy and solubility is less well defined for alkali salts of oxoanions; in particular, the anomalous solubilities of some highly charged oxoanions including carbonate [6,7], which is further complicated by protonation in solution.

We define anomalous solubility as increased solubility with increased ion-pairing, while normal solubility is the opposite. As an initial approximation, oxoanions and POMs with low charge density exhibit the typical solubility trend (less soluble with increasing alkali countercation size), whereas those with high charge densities exhibit the reversed, atypical solubility trend. Some common POMs and simple oxoanions, their charge densities (described here as cluster charge divided by number of cluster atoms), and observed solubility trends as alkali salts are summarized in Table 1 and below we provide a qualitative explanation of these opposite solubility trends. We surmise that oxo species with higher charge densities exhibit this atypical trend due to their ability to disrupt the relatively weakly bound hydration spheres of larger alkali cations (i.e., Rb^+^ and Cs^+^), allowing for contact ion pairing. Because of the high POM/oxoanion charge, the alkali is strongly associated and therefore does not bridge to other oxoanions, suppressing aggregation and precipitation [8]. On the other hand, the high charge density of the same POMs/oxoanions does not completely satisfy bonding of the smaller and more charge-dense alkali cations (i.e., Li^+^ and Na^+^), therefore these alkalis bridge POM/oxoanions in solution, initiating precipitation. Considering the low-charge density POMs, they may also form contact ion-pairs with larger alkalis, but these alkalis are not as strongly associated to a single POM/oxoanion. Rather they bridged two or more of these anions in solution, initiating precipitation. Finally the low-charge density POMs likely cannot penetrate the large hydration sphere of the charge-dense alkalis (Li^+^, Na^+^), so there is no interaction in solution and solubility is retained.

Understanding the governing principles of solubility is important because it yields more complete models for thermodynamic calculations of aqueous speciation and ion association. Additionally, the solubility and association of ions in water affects their adsorption onto metal oxide surfaces [9], and knowledge of ion association can be used to fine-tune the electronic properties and chemical reactivities of catalysts, as well as design ion-specific sorbents [10,11,12].

Polyoxometalates (POMs), nanoscale molecular oxoanions of Group V and VI metals in their highest oxidation states, can generally be synthesized with any alkali cation, thus providing an excellent model for ion-pairing in water and at metal oxide surfaces [15,22]. These “molecular metal oxides” exhibit a wide range of aqueous behaviors that depend on their metal centers and, crucially, their countercations [23]. The isostructural and isovalent hexaniobate ([Nb_6_O_19_]^8-^, Nb${}_{6}$) and hexatantalate ([Ta_6_O_19_]^8-^, Ta${}_{6}$) POMs exhibit very similar pH stabilities, and aqueous solubilities with respect to alkali countercations [16,24,25]. In particular, Cs^+^ undergoes significant ion-pairing with these POMs, which increases their solubilities due to each Cs^+^ more favorably interacting with a single cluster rather than coordinating to multiple clusters, wherein binding to a single cluster inhibits aggregation and precipitation, even at high concentration [8]. However, subtle differences have been found in their degrees of ion association with Cs^+^. For example, Ta${}_{6}$ undergoes greater ion-pairing than Nb${}_{6}$ with Cs^+^ in dilute solutions in neat water, even at low concentration. This was attributed to the presence of relativistic effects in Ta${}_{6}$, which results in the orbital interaction term having a larger contribution to the total bonding energy [8]. In other words, electrostatics alone are insufficient to describe cation association in the presence of Group V POMs, and covalent bonding between the alkalis and oxo ligands may also be considered, in order to arrive at a complete description of solution behavior.

## 2. Results & Discussion

### 2.1. Solid-State Calorimetry of Ta${}_{6}$ Salts

**Figure 1:**
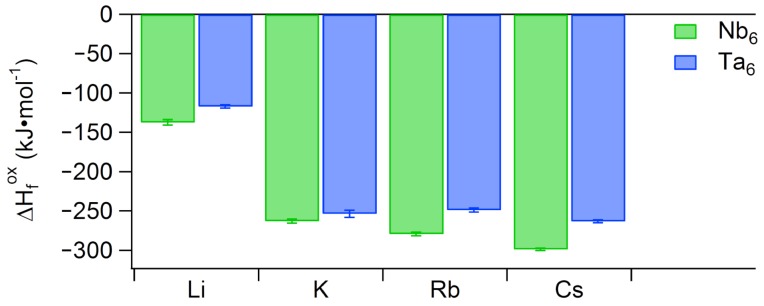
Solid-state $\Delta H_{f}^{ox}$ (enthalpy of formation from oxides, kJ (mol-Nb)${}^{-1}$) values for alkali salts of Ta_6_ (this study) and Nb_6_ (prior study) [26].

We previously conducted high-temperature oxide decomposition calorimetric studies on alkali (Li^+^, K^+^, Rb^+^, Cs^+^) salts of Nb${}_{6}$ [26]. Upon dropping pellets made from crystalline samples into molten sodium molybdate at 700 ${}^{\circ }C$, we ascertained a trend of increasingly exothermic enthalpy of formation from oxides ($\Delta H_{f}^{ox}$) with respect to alkali countercation size. To parallel this previous study, we performed measurements on the same alkali salts of Ta${}_{6}$. Although the Na_8_[Ta_6_O_19_] salt can be synthesized, it was not studied because the analogous Nb${}_{6}$ salt is not readily obtained. Rather, Na_7_[HNb_6_O_19_] is the common form [27]. The measured $\Delta H_{f}^{ox}$ is less exothermic for each alkali Ta_6_ analogue, compared to the Nb_6_ analogue (Figure 1). We rationalize this as mixing of Ta${}_{5d}$ and O${}_{2p}$ orbitals in Ta${}_{6}$ being poorer than the mixing between Nb${}_{4d}$ and O${}_{2p}$ in Nb${}_{6}$ [8,18], decreasing the thermodynamic stability of the Ta${}_{6}$ cluster in comparison to the Nb${}_{6}$ cluster. As with the observed trend for hexaniobate, the Li^+^ salt of Ta${}_{6}$ exhibits a far less exothermic (by more than a factor of two) $\Delta H_{f}^{ox}$ than the larger alkali salts. This is explained by the structure of lithium hexaniobate [28,29]. The hexaniobate lattice contains adamantane-like Li-water clusters, which prevents extensive direct bonding between Li^+^ and Nb${}_{6}$. A high-resolution single-crystal structure of Li–Ta${}_{6}$ has never been possible, due to poor crystal quality and perhaps disorder of the hydrated lithium in the lattice. We surmise that Li–Ta${}_{6}$ is not isostructural with the Li–Nb${}_{6}$ analogue (Li_8_Nb_6_O_19_·22H_2_O) due to the lack of a reported single-crystal structure for lithium hexatantalate [30,31]. However, Li is generally bonded to water in hydrated salt lattices, and this is reflected in its less exothermic formation enthalpy, compared to the salts of the heavier alkalis. Unlike Nb${}_{6}$, the K^+^, Rb^+^, and Cs^+^ salts of Ta${}_{6}$ do not exhibit a strict trend of increasing $\Delta H_{f}^{ox}$ (Figure 1). Instead, the three larger alkali salts are more similar in their $\Delta H_{f}^{ox}$. This suggests that Ta${}_{6}$ is slightly `less selective’ in its solid-state ion association than Nb${}_{6}$.

### 2.2. Dissolution Energy of Ta${}_{6}$ Salts

**Figure 2:**
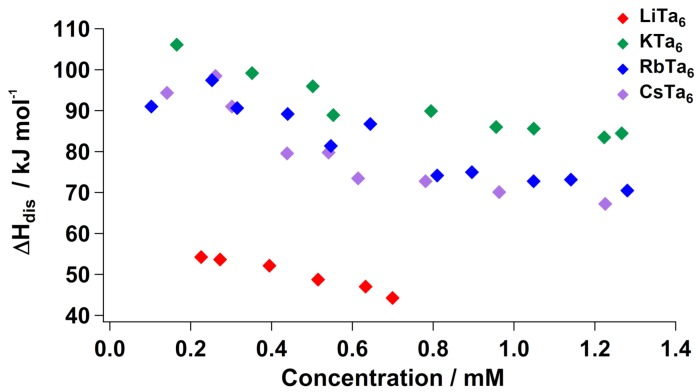
Enthalpy of aqueous dissolution ($\Delta H_{dis}$) of Li–Ta_6_, K–Ta_6_, Rb–Ta_6_ and Cs–Ta_6_ in water, normalized for lattice water (i.e., representing dissolution of the dehydrated forms Li_8_[Ta_6_O_19_], K_8_[Ta_6_O_19_], Rb_8_[Ta_6_O_19_] and Cs_8_[Ta_6_O_19_]; the hydrated enthalpies are provided in Tables S5–S9).

While high-temperature calorimetry provides information about the stability of the clusters and their association with lattice species, solution calorimetry elucidates hydration enthalpies of the ions upon dissolution, as well as their solid-state lattice interactions. Enthalpy of dissolution ($\Delta H_{dis}$) at room temperature of the Nb${}_{6}$ salts in neat water showed that the Cs-salt has greater concentration dependence in its dissolution enthalpy compared to the other alkali salts. By extension, the degree of structural change became less extensive at higher concentrations, indicating that the Cs^+^ underwent maximum ion-pairing with Nb_6_, even at the low concentrations (<2 mM). Analogous measurements of Ta_6_ salts also showed $\Delta H_{dis}$ concentration dependence for each alkali salt (Figure 2). Li^+^ again cannot be easily compared to the larger alkalis, due to its far more extensive coordination to water molecules in the solid state. This is reflected in its overall lower values of $\Delta H_{dis}$ compared to the other three alkali salts, meaning it is not necessary to disrupt extensive bonding between the clusters and alkalis to dissolve them, which is an endothermic process. However, the steeper slope of the Cs–Ta${}_{6}$ trend was matched by the Rb^+^ salt, with K^+^ having a smaller slope. This indicates that rubidium and cesium undergo similar degrees of ion-pairing with Ta${}_{6}$ in solution, which is different from the observed Nb${}_{6}$ trend, in which the cesium salt clearly had the greatest dependence on concentration. Like the high-temperature calorimetry, this too indicates that Ta${}_{6}$ is `less selective’ in its ion association than Nb${}_{6}$ (in both solution and solid state). On the other hand, it has been noted that Nb${}_{6}$ is more basic than Ta${}_{6}$, meaning it protonates upon dissolution in water, at the bridging oxos sites [32,33]. Since protonation is an endothermic process that is not equal between the Ta${}_{6}$ and Nb${}_{6}$, it is difficult to compare the dissolution enthalpy with exact certainty.

**Figure 3:**
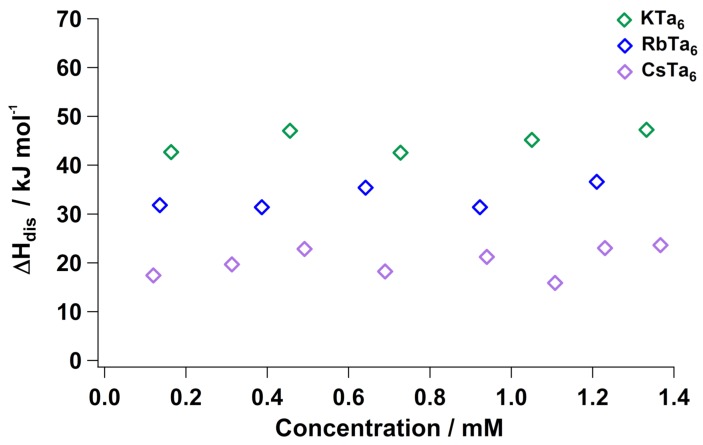
Enthalpy of aqueous dissolution ($\Delta H_{dis}$) of Li–Ta_6_, K–Ta_6_, Rb–Ta_6_ and Cs-Ta_6_ in their parent hydroxide (1 M) solutions, normalized for lattice water. The hydrated enthalpies are provided in Tables S10–S13.

We dissolved the K, Rb and Cs Ta${}_{6}$ salts in 1 molar solutions of each alkali cation’s parent hydroxide (Figure 3). The Li^+^ salt was omitted due to its insolubility in LiOH. Like Nb${}_{6}$, alkali salts of Ta${}_{6}$ do not have a concentration dependence when dissolved in base, due to a lack of cluster protonation and the excess cations in solution forcing the “maximally associated” state at all concentrations. The generally more exothermic dissolution enthalpies can be related to the lack of protonation of the clusters with high concentration of base. There is a distinct trend in $\Delta H_{dis}$ with respect to alkali cation size; with Cs–Ta${}_{6}$ being most exothermic, followed by Rb-Ta_6_ and then K–Ta${}_{6}$. This trend suggested that less energy is required to dissociate the Cs–Ta${}_{6}$ lattice adequately to achieve dissolution, compared to the K and Rb analogues. This is exactly consistent with ion-pairing persisting in solution: increasing K–Ta${}_{6}$< Rb–Ta${}_{6}$< Cs–Ta${}_{6}$. The solutions remain monodisperse and no appreciable amount of impurities form with added solid Ta${}_{6}$, accounted for by previous small-angle X-ray scattering studies of hexatantalate in 1 molar parent hydroxide solution [24]. We also do not expect any impurities or cluster alteration upon dissolution in hydroxide solutions because the hexatantalate form is most stable in conditions of high concentration hydroxide, confirming that the flatlining of $\Delta H_{dis}$ is strictly from maximal A–Ta${}_{6}$ ion-pairing.

**Figure 4:**
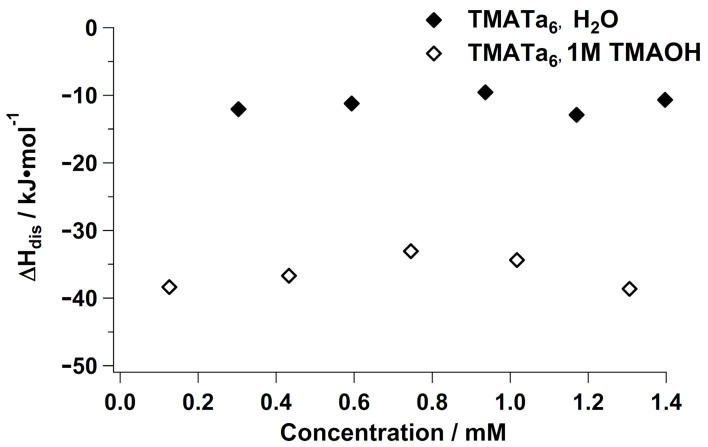
Enthalpy of dissolution of TMA–Ta_6_ in neat water and in 1M TMAOH.

Finally, we compared the $\Delta H_{dis}$ of the tetramethylammonium salt of Ta${}_{6}$ ([(CH_3_)_4_N]_6_H_2_O_19_·21H_2_O, TMA–Ta${}_{6}$) in water and in 1 molar TMAOH to the previously measured Nb analogue (Figure 4). As with the analogous TMA–Nb${}_{6}$ study, $\Delta H_{dis}$ does not have a concentration dependence in either solution environment due to the lack of ion association between TMA^+^ and Ta${}_{6}$. However, $\Delta H_{dis}$ is less exothermic for TMA–Ta${}_{6}$ (∼−10 ${kJ\;\mathrm{mol}}^{-1}$) than it is for TMA–Nb${}_{6}$ (∼−40 ${kJ\;\mathrm{mol}}^{-1}$) in water. As described above, this is because Ta${}_{6}$ is a weaker base than Nb${}_{6}$ [25], resulting in a lesser degree of deprotonation, consistent with fewer exothermic events occurring in solution. The difference in $\Delta H_{dis}$ between TMA–Ta${}_{6}$ and TMA–Nb${}_{6}$ is greatly enhanced in 1M TMAOH (∼−35 ${kJ\;\mathrm{mol}}^{-1}$ and (∼−35 ${kJ\;\mathrm{mol}}^{-1}$, respectively). This is due to the tendency for TMA–Nb${}_{6}$ to form oligomeric chains in neat water, whereas TMA–Ta${}_{6}$ typically forms dimers [24]. Thus, the vast difference in exothermic dissolution is further explained by the degree of hydrolysis of the respective assemblies—a far greater number of exothermic events occur in dissociating the Nb_6_ chains than in dissociating Ta${}_{6}$ dimers. The $\Delta H_{dis}$ is nonetheless more exothermic for TMA–Ta${}_{6}$ in 1M TMAOH than it is in neat water due to the disassembly of these dimers.

## 3. Materials and Methods

### 3.1. Determination of Drop Solution Enthalpies

$\Delta H_{ds}$ was measured in a custom-made isoperibol Tian-Calvet twin microcalorimeter. Pellets of about 5 mg were loosely pressed, weighed, and dropped from room temperature into 3Na_2_O·4MoO_3_ molten solvent at 702 ${}^{\circ }C$. The calorimeter assembly was washed with oxygen at 43 ${mL\;\min}^{-1}$. Oxygen was bubbled through the solvent at 4.5 ${mL\;\min}^{-1}$ to aid dissolution, evolve water vapor, and to maintain oxidizing conditions. The calorimeter was calibrated against the heat content of 5 mg pellets of high-purity Al_2_O_3_ (99.997%, Alfa Aesar) dropped into an empty crucible.

### 3.2. Determination of Room Temperature Dissolution Enthalpies

$\Delta H_{dis}$ was measured using a CSC 4400 isothermal microcalorimeter operated at 25 ${}^{\circ }C$. About 10–25 mg of each sample was hand pressed into a pellet and dropped one at a time into 25.0 g of H_2_O. Each experiment was repeated in 1 M AOH (A = K, Rb, Cs, TMA) for the respective clusters. The lithium salt was omitted due to its insolubility.

The calorimeter was calibrated by dissolving 15 mg pellets of KCl in water with stirring at 25 ${}^{\circ }C$. Hydrous and anhydrous cluster dissolution enthalpy values in water are reported in Tables S5–S9 and parent hydroxide values are reported in Tables S10–S13.

Enthalpies of anhydrous clusters were found by subtracting the enthalpy of dissolution of lattice water (0.4 ${kJ\;\mathrm{mol}}^{-1}$) [34] and then adjusting for the relative molar weights of the hydrated and anhydrous clusters, namely:

(1) $$\Delta H_{dis,anhydrous}=\left(\Delta H_{dis,hydrated}-\left(0.4{\;kJ\;\mathrm{mol}}^{-1}\right)\left(n_{H_{2}O}\right)\right)\frac{\mathrm{MW}(\mathrm{Anhydrous}\;\mathrm{Cluster})}{\mathrm{MW}(\mathrm{Hydrated}\;\mathrm{Cluster})}$$

### 3.3. Thermogravimetric Analysis

Thermogravimetry was used to determine number of lattice waters per cluster. These experiments were performed on a Setaram LabSys Evo. The sample was heated in an alumina crucible from 25 ${}^{\circ }C$ to 600 ${}^{\circ }C$ in an inert atmosphere at 10 ${}^{\circ }C\;\min^{-1}/$. The total weight loss was determined at the temperature where weight % reached a minimum (Figures S5–S8).

### 3.4. Syntheses

#### 3.4.1. Alkali Salts

The larger alkali salts of [Ta_6_O_19_]^8-^ were synthesized by refluxing a ≈40 mM solution of their peroxotantalate analogues (A_3_Ta(O_2_)_4_; A = K, Rb, Cs) in concentrated parent alkali hydroxide solution for 4 h, except for the lithium salt, which was formed by metathesis of the potassium salt in 1 M LiOH solution. The peroxotantalates were formed by adding 4.6 g TaCl_5_ to 40 mL of cold 30% (*w/w*) H_2_O_2_ solution, adding concentrated AOH solutions (A = K, Rb, Cs), and precipitating with ethanol. Full syntheses are described in a prior publication [27].

#### 3.4.2. Tetramethylammonium Salt

Tetramethylammonium hexatanatalate ([(CH_3_)_4_N]_6_H_2_Ta_6_O_19_) was synthesized by adding 1.32 g of (NH_4_)_3_Ta(O_2_)_4_ to 8.25 mL of 1.4 M tetramethylammonium hydroxide and refluxing for 5 h. The solution was then microfiltered, 40 mL isopropyl alcohol was added, and then the resulting solution was centrifuged to yield a small denser layer containing the product. The supernatant was discarded and further addition of 30 mL isopropyl alcohol yielded a white precipitate, which was washed with more isopropyl alcohol and oven-dried under vacuum at 60 ${}^{\circ }C$ [24].

## 4. Conclusions

This study represents a rare quantitative evaluation of combined high temperature and aqueous dissociation energy of water-soluble metal-oxo clusters, elucidating the correlation between ion-association and acid-base behavior in dissolution of cluster salts, as well as the stability of the clusters. Experimentally determined enthalpy of formation of the Nb and Ta hexametalate POMs showed lower thermodynamic stability of the Ta-analogues compared to the Nb-analogues, regardless of the alkali counterion. This is consistent with the slightly larger ionic radius of Ta^5+^ than Nb^5+^, and corresponding weaker M–O bonds within the POM. While all cluster salts exhibit negative enthalpy of formation from oxides, there is a periodic trend of increasingly exothermic enthalpy with increasing alkali size. Notably fewer negative enthalpies are measured for the Li-salts compared to the heavier congeners, due to minimal cluster-cation association within the lattice (lower lattice energy). Aqueous dissolution enthalpy is the net sum of both endothermic and exothermic processes including protonation and/or deprotonation of the oxoanion POMs, dissociation of ions, and hydration of ions. With the various solutions (neat water and parent hydroxides) and various countercations (alkalis and tetramethylammounium, TMA) for the Nb and Ta hexametalates, we can conclude the following about cation-oxoanion lattice energetics. First, protonation of oxoanions such as POMs stabilize lattices even more than the strongly associated heavier alkali cations. Second, solubility of oxoanions cannot be described as inversely correlated with their lattice energy: we observed both the most negative and the most positive lattice energies for the two most soluble salts, respectively with TMA^+^ and with Cs^+^ counterions. This means that lattice energy, as classically described by the Born-Haber cycle, does not necessarily predict solubility of oxoanion salts, or lattices containing aqua species.

## Figures and Tables

**Table 1:** Charge densities, defined as the total charge divided by the number of non-hydrogen atoms, and solubility trends with respect to alkali countercations of several prominent oxoanions and POMs.

Species	# of Non-Hydrogen Atoms	Charge Density	Solubility Trend
[PO_4_]^3-^	5	0.6	Anomalous [13]
[CO_3_]^2-^	4	0.5	Anomalous [13]
[ClO_4_]^-^	5	0.2	Normal [14]
[Nb_6_O_19_]^8-^	25	0.32	Anomalous [15]
[Ta_6_O_19_]^8-^	25	0.32	Anomalous [16]
[SiNb_12_O_40_]^16-^	53	0.30	Anomalous [16]
[GaNb_18_O_54_]^15-^	73	0.21	Anomalous [17]
[Nb_4_W_2_O_19_]^6-^	25	0.24	Anomalous [18]
[Nb_2_W_4_O_19_]^4-^	25	0.16	Normal [18]
[W_6_O_19_]^2-^	25	0.08	Normal *
[SiW_12_O_40_]^4-^	53	0.075	Normal [19]
[P_2_W_18_O_62_]^6-^	82	0.073	Normal [20]
[PW_12_O_40_]^3-^	53	0.057	Normal [19]

* Only soluble in organic solvents [21].

## Supplementary Materials

The following are available online. Thermochemical Cycles (with oxide drop solution enthalpies taken from previous studies) [35], values for hydrated (non-normalized for water) enthalpies of dissolution, thermogravimetric analyses, and energy dispersive X-ray spectra are available as Supplementary Information.

## Abbreviations

The following abbreviations are used in this manuscript:

POM	Polyoxometalate
Nb${}_{6}$	Hexaniobate
Ta${}_{6}$	Hexatantalate
TMA	Tetramethylammonium
