# Supplementary material for: Thermochemical Measurements of Alkali Cation Association to Hexatantalate
Source: Molecules. 2018 Sep 24;23(10):2441. doi: 10.3390/molecules23102441 (PMC6222682; doi:10.3390/molecules23102441)
Supplement: Supplementary file 1 [file molecules-23-02441-s001.zip › molecules-360639-SI.pdf]

Thermochemical measurements of alkali cation association to hexatantalate – Supplementary Information

Dylan Sures [1,2*], G.P. Nagabhushana[3], Alexandra Navrotsky[3], and May Nyman [1,*]

Contents

S1 Drop Solution Enthalpies **S2**

S2 Thermochemical Cycles **S3**

S3 Room Temperature Dissolution Enthalpies **S5**
S3.1 Tables of Aqueous Dissolution Enthalpies S6
S3.2 Tables of 1M Parent Hydroxide Dissolution Enthalpies S8

S4 Supplementary Characterization **S9**
S4.1 Energy Dispersive X-ray Analysis (EDX) S9
S4.2 Thermogravimetric Analysis S13

[1]Department of Chemistry, Oregon State University, Corvallis, Oregon 97331-4003, United States

[2]Department of Chemistry, University of California, Davis, One Shields Avenue, Davis, California 95616, United States

[3]Peter A. Rock Thermochemistry Laboratory and NEAT ORU, University of California Davis, Davis, CA 95616, United States

*Email: sures@ucdavis.edu, may.nyman@oregonstate.edu

S1 Drop Solution Enthalpies

Table S1: Drop solution enthalpy of $Li_8Ta_6O_{19}$

Mass (mg)	ΔH_{ds} (kJ mol^{-1})
4.73	1860.21
3.85	1853.85
3.75	1839.61
3.47	1856.16
3.00	1855.44
3.69	1859.97
2.31	1862.38
3.22	1856.14
Average:	1855.47 ± 4.97
-18 H_2O	-1242.00
**Final**:	**613.47 ± 4.97**

Table S2: Drop solution enthalpy of $K_8Ta_6O_{19}$

Mass (mg)	ΔH_{ds} (kJ mol^{-1})
4.65	1586.34
4.91	1672.26
5.41	1603.25
5.13	1586.34
7.87	1647.62
4.29	1635.12
5.39	1665.30
6.97	1627.86
Average	1628.01 ± 23.71
-16 H_2O	-1104.00
**Final:**	**514.01 ± 23.71**

Table S3: Drop solution enthalpy of $Rb_8Ta_6O_{19}$

Mass (mg)	ΔH_{ds} (kJ mol^{-1})
3.92	1406.56
5.74	1403.56
4.31	1388.76
5.38	1389.67
5.33	1379.51
7.61	1427.34
6.88	1401.91
4.40	1401.85
Average:	1399.89 ± 10.22
-14 H_2O	-966.00
**Final**:	**433.89 ± 10.22**

Table S4: Drop solution enthalpy of $Cs_8Ta_6O_{19}$

Mass (mg)	ΔH_{ds} (kJ mol^{-1})
4.95	1415.26
5.69	1421.01
4.58	1409.12
4.35	1427.62
5.70	1426.25
7.83	1413.61
4.20	1423.81
4.02	1422.82
Average:	1419.94 ± 4.65
-14 H_2O	-966.00
**Final**:	**453.94 ± 4.65**

S2 Thermochemical Cycles

Lithium Hexatantalate

The formation enthalpy of $Li_8Ta_6O_{19}$ from constituent binary oxides is -117.2 kJ/mol Ta. The correction of 69 kJ/mol H_2O for lattice water has been applied as seen in Table S1.

$$\frac{1}{6}\{Li_8Ta_6O_{19}\}(\text{xl, 25°C}) \rightarrow \frac{2}{3}Li_2O(\text{soln, 702°C}) + \frac{1}{2}Ta_2O_5(\text{soln, 702°C}) \tag{S1}$$
$$\Delta H_1 = \Delta H_{ds} = 102.25 \pm 0.83\text{kJ/mol Ta}$$

$$Ta_2O_5(\text{s, 25°C}) \rightarrow Ta_2O_5(\text{soln, 702°C} \tag{S2}$$
$$\Delta H_2 = \Delta H_{ds}(Ta_2O_5) = 90.41 \pm 2.50\text{kJ/mol Ta}$$

$$Li_2O(\text{xl, 25°C}) \rightarrow Li_2O(\text{soln, 702°C}) \tag{S3}$$
$$\Delta H_3 = \Delta H_{ds}(Li_2O) = -90.3 \pm 2.5\text{kJ/mol Ta}$$

$$\frac{2}{3}Li_2O + \frac{1}{2}Ta_2O_5\ (\text{xl, 25°C}) \rightarrow \frac{1}{6}\{Li_8Nb_6O_{19}\}\ (\text{xl, 25°C}) \tag{S4}$$
$$\Delta H_4 = \Delta H_f^{ox}(Li_8Ta_6O_{19}) = -\Delta H_1 + \frac{1}{2}\Delta H_2 + \frac{2}{3}\Delta H_3 = -117.2 \pm 2.1\text{kJ/mol Ta}$$

Potassium Hexatantalate

The formation enthalpy of $K_8Ta_6O_{19}$ from constituent binary oxides is -253.7 kJ/mol Ta. The correction of 69 kJ/mol H_2O for lattice water has been applied as seen in Table S2.

$$\frac{1}{6}\{K_8Ta_6O_{19}\}(\text{xl, 25°C}) \rightarrow \frac{2}{3}K_2O(\text{soln, 702°C}) + \frac{1}{2}Ta_2O_5(\text{soln, 702°C}) \tag{S5}$$
$$\Delta H_5 = \Delta H_{ds} = 72.32 \pm 3.95\text{kJ/mol Ta}$$

$$K_2O(\text{xl, 25°C}) \rightarrow K_2O(\text{soln, 702°C}) \tag{S6}$$
$$\Delta H_6 = \Delta H_{ds}(K_2O) = -318.0 \pm 3.1\text{kJ/mol Ta}$$

$$\frac{2}{3}K_2O + \frac{1}{2}Ta_2O_5\ (\text{xl, 25°C}) \rightarrow \frac{1}{6}\{K_8Ta_6O_{19}\}\ (\text{xl, 25°C}) \tag{S7}$$
$$\Delta H_7 = \Delta H_f^{ox}(K_8Ta_6O_{19}) = -\Delta H_5 + \frac{1}{2}\Delta H_2 + \frac{2}{3}\Delta H_6 = -253.7 \pm 4.4\text{kJ/mol Ta}$$

Rubidium Hexatantalate

The formation enthalpy of $Rb_8Ta_6O_{19}$ from constituent binary oxides is -248.9 kJ/mol Ta. The correction of 69 kJ/mol H_2O for lattice water has been applied as seen in Table S3.

$$\frac{1}{6}\{Rb_8Ta_6O_{19}\}(\text{xl, 25°C}) \rightarrow \frac{2}{3}Rb_2O(\text{soln, 702°C}) + \frac{1}{2}Ta_2O_5(\text{soln, 702°C}) \tag{S8}$$

$$\Delta H_8 = \Delta H_{ds} = 85.67 \pm 1.70\text{kJ/mol Ta}$$

$$Rb_2O(\text{xl, 25°C}) \rightarrow Rb_2O(\text{soln, 702°C}) \tag{S9}$$

$$\Delta H_9 = \Delta H_{ds}(Rb_2O) = -332.6 \pm 2.2\text{kJ/mol Ta}$$

$$\frac{2}{3}Rb_2O + \frac{1}{2}Ta_2O_5\ (\text{xl, 25°C}) \rightarrow \frac{1}{6}\{Rb_8Ta_6O_{19}\}\ (\text{xl, 25°C}) \tag{S10}$$

$$\Delta H_{10} = \Delta H_f^{ox}(Rb_8Ta_6O_{19}) = -\Delta H_8 + \frac{1}{2}\Delta H_2 + \frac{2}{3}\Delta H_9 = -248.9 \pm 2.7\text{kJ/mol Ta}$$

Cesium Hexatantalate

The formation enthalpy of $Cs_8Ta_6O_{19}$ from constituent binary oxides is -263.1 kJ/mol Ta. The correction of 69 kJ/mol H_2O for lattice water has been applied as seen in Table S4.

$$\frac{1}{6}\{Cs_8Ta_6O_{19}\}(\text{xl, 25°C}) \rightarrow \frac{2}{3}Cs_2O(\text{soln, 702°C}) + \frac{1}{2}Ta_2O_5(\text{soln, 702°C}) \tag{S11}$$

$$\Delta H_{11} = \Delta H_{ds} = 75.66 \pm 0.81\text{kJ/mol Ta}$$

$$Cs_2O(\text{xl, 25°C}) \rightarrow Cs_2O(\text{soln, 702°C}) \tag{S12}$$

$$\Delta H_{12} = \Delta H_{ds}(Cs_2O) = -348.9 \pm 1.7\text{kJ/mol Ta}$$

$$\frac{2}{3}Cs_2O + \frac{1}{2}Ta_2O_5\ (\text{xl, 25°C}) \rightarrow \frac{1}{6}\{Cs_8Ta_6O_{19}\}\ (\text{xl, 25°C}) \tag{S13}$$

$$\Delta H_{13} = \Delta H_f^{ox}(Cs_8Ta_6O_{19}) = -\Delta H_{11} + \frac{1}{2}\Delta H_2 + \frac{2}{3}\Delta H_{12} = -263.1 \pm 1.9\text{kJ/mol Ta}$$

Where ΔH_{ds} are drop solution enthalpies under oxygen bubbling.

S3 Room Temperature Dissolution Enthalpies

Dissolution enthalpies of hydrated hexatantalate clusters in water

$Li_8Ta_6O_{19} \cdot 18H_2O \longrightarrow 8\,Li^+ + [H_xTa_6O_{19}]^{(8-x)-} + (18\text{-}x)H_2O + xOH^-$
$K_8Ta_6O_{19} \cdot 16H_2O \longrightarrow 8\,K^+ + [H_xTa_6O_{19}]^{(8-x)-} + (16\text{-}x)H_2O + xOH^-$
$Rb_8Ta_6O_{19} \cdot 14H_2O \longrightarrow 8\,Rb^+ + [H_xTa_6O_{19}]^{(8-x)-} + (14\text{-}x)H_2O + xOH^-$
$Cs_8Ta_6O_{19} \cdot 14H_2O \longrightarrow 8\,Cs^+ + [H_xTa_6O_{19}]^{(8-x)-} + (14\text{-}x)H_2O + xOH^-$
$(TMA)_6H_2Ta_6O_{19} \cdot 21H_2O \longrightarrow 6\,TMA^+ + [H_{4+x}(Ta_6O_{19})_2]^{(12-x)} + (21\text{-}x)H_2O + xOH^-$

Dissolution enthalpies of anhydrous hexatantalate clusters in water

$Li_8Ta_6O_{19} + xH_2O \longrightarrow 8\,Li^+ + [H_xTa_6O_{19}]^{(8-x)-} + xOH^-$
$K_8Ta_6O_{19} + xH_2O \longrightarrow 8\,K^+ + [H_xTa_6O_{19}]^{(8-x)-} + xOH^-$
$Rb_8Ta_6O_{19} + xH_2O \longrightarrow 8\,Rb^+ + [H_xTa_6O_{19}]^{(8-x)-} + xOH^-$
$Cs_8Ta_6O_{19} + xH_2O \longrightarrow 8\,Cs^+ + [H_xTa_6O_{19}]^{(8-x)-} + xOH^-$
$(TMA)_6H_2Ta_6O_{19} + xH_2O \longrightarrow 6\,TMA^+ + [H_{4+x}(Ta_6O_{19})_2]^{(12-x)} + xOH^-$

Dissolution enthalpies of hydrated hexatantalate clusters in 1M parent hydroxide

$Li_8Ta_6O_{19} \cdot 18H_2O \longrightarrow 8\,Li^+ + [Ta_6O_{19}]^{8-} + 18H_2O$
$K_8Ta_6O_{19} \cdot 18H_2O \longrightarrow 8\,K^+ + [Ta_6O_{19}]^{8-} + 18H_2O$
$Rb_8Ta_6O_{19} \cdot 18H_2O \longrightarrow 8\,Rb^+ + [Ta_6O_{19}]^{8-} + 18H_2O$
$Cs_8Ta_6O_{19} \cdot 18H_2O \longrightarrow 8\,Cs^+ + [Ta_6O_{19}]^{8-} + 18H_2O$
$(TMA)_6H_2Ta_6O_{19} \cdot 21H_2O + 2\,OH^- \longrightarrow 6\,TMA^+ + [Ta_6O_{19}]^{8-} + 23H_2O$

Dissolution enthalpies of anhydrous hexatantalate clusters in 1M parent hydroxide

$Li_8Ta_6O_{19} \longrightarrow 8\,Li^+ + [Ta_6O_{19}]^{8-}$
$K_8Ta_6O_{19} \longrightarrow 8\,K^+ + [Ta_6O_{19}]^{8-}$
$Rb_8Ta_6O_{19} \longrightarrow 8\,Rb^+ + [Ta_6O_{19}]^{8-}$
$Cs_8Ta_6O_{19} \longrightarrow 8\,Li^+ + [Cs_6O_{19}]^{8-}$
$(TMA)_6H_2Ta_6O_{19} + 2\,OH^- \longrightarrow 6\,TMA^+ + [Ta_6O_{19}]^{8-} + 2\,H_2O$

S3.1 Tables of Aqueous Dissolution Enthalpies

Table S5: Lithium Hexatantalate Dissolution Enthalpies in Water

Concentration (M $\times 10^5$)	Dissolution Enthalpy (ΔH_{dis} / kJ mol^{-1})	Anhydrous Dissolution Enthalpy (ΔH_{dis} / kJ mol^{-1})
22.59	73.61	54.24
27.27	72.90	53.67
39.49	71.06	52.17
51.54	66.91	48.77
63.29	64.77	47.02
69.94	61.39	44.27

Table S6: Potassium Hexatantalate Dissolution Enthalpies in Water

Concentration (M $\times 10^5$)	Dissolution Enthalpy (ΔH_{dis} / kJ mol^{-1})	Anhydrous Dissolution Enthalpy (ΔH_{dis} / kJ mol^{-1})
16.56	130.46	106.10
35.20	122.37	99.18
50.19	118.60	95.94
55.29	110.35	88.91
79.44	111.50	89.89
95.57	106.98	86.02
104.90	106.52	85.63
122.31	104.02	83.49
126.64	101.47	81.31

Table S7: Rubidium Hexatantalate Dissolution Enthalpies in Water

Concentration (M $\times 10^5$)	Dissolution Enthalpy (ΔH_{dis} / kJ mol^{-1})	Anhydrous Dissolution Enthalpy (ΔH_{dis} / kJ mol^{-1})
10.25	107.69	91.02
25.29	114.87	97.42
31.49	107.27	90.64
44.00	105.64	89.20
65.45	102.88	86.73
80.98	88.78	74.16
89.57	89.69	74.97
104.87	87.23	72.78
114.09	87.66	73.16
128.03	84.66	70.49

Table S8: Cesium Hexatantalate Dissolution Enthalpies in Water

Concentration (M $\times 10^5$)	Dissolution Enthalpy (ΔH_{dis} / kJ mol^{-1})	Anhydrous Dissolution Enthalpy (ΔH_{dis} / kJ mol^{-1})
14.14	109.64	94.34
26.14	114.21	98.48
31.09	105.95	91.00
43.84	93.33	79.56
54.10	93.60	79.79
61.43	86.61	73.46
78.12	85.84	72.76
96.32	82.94	70.13
122.55	79.77	67.25

Table S9: Tetramethylammonium Hexatantalate Dissolution Enthalpies in Water

Concentration (M $\times 10^5$)	Dissolution Enthalpy (ΔH_{dis} / kJ mol^{-1})	Anhydrous Dissolution Enthalpy (ΔH_{dis} / kJ mol^{-1})
30.32	-5.02	-12.04
59.36	-4.17	-11.20
93.65	-2.53	-9.55
117.01	-5.87	-12.89
139.70	-3.65	-10.68

S3.2 Tables of 1M Parent Hydroxide Dissolution Enthalpies

Table S10: Potassium Hexatantalate Dissolution Enthalpies in 1M KOH

Concentration (M $\times 10^5$)	Dissolution Enthalpy (ΔH_{dis} / kJ mol^{-1})	Anhydrous Dissolution Enthalpy (ΔH_{dis} / kJ mol^{-1})
16.34	56.329	42.70
45.57	61.43	47.06
72.78	56.17	42.56
105.07	59.23	45.18
133.24	61.68	47.28

Table S11: Rubidium Hexatantalate Dissolution Enthalpies in 1M RbOH

Concentration (M $\times 10^5$)	Dissolution Enthalpy (ΔH_{dis} / kJ mol^{-1})	Anhydrous Dissolution Enthalpy (ΔH_{dis} / kJ mol^{-1})
13.57	41.31	31.84
38.66	40.82	31.40
64.17	40.81	35.41
92.30	46.66	31.39
121.00	46.02	36.61

Table S12: Cesium Hexatantalate Dissolution Enthalpies in 1M CsOH

Concentration (M $\times 10^5$)	Dissolution Enthalpy (ΔH_{dis} / kJ mol^{-1})	Anhydrous Dissolution Enthalpy (ΔH_{dis} / kJ mol^{-1})
11.99	24.82	17.43
31.28	27.32	19.70
49.15	30.80	22.85
68.96	25.71	18.24
93.97	29.01	21.23
110.76	21.05	14.01
123.02	30.99	23.02
136.68	31.66	23.63

Table S13: TMA Hexaniobate Dissolution Enthalpies in 1M TMAOH

Concentration (M $\times 10^5$)	Dissolution Enthalpy ΔH_{dis} / kJ mol^{-1}	Anhydrous Dissolution Enthalpy ΔH_{dis} / kJ mol^{-1}
12.62	-40.39	-38.37
43.26	-38.26	-36.69
74.52	-33.63	-33.05
101.71	-35.31	-34.37
130.60	-40.72	-38.63

S4 Supplementary Characterization

S4.1 Energy Dispersive X-ray Analysis (EDX)

EDX Spectra were obtained from a Quanta 600F instrument (FEI) operating at an accelerating voltage of 20 kV. Measurements were taken five times throughout each sample to ensure accuracy.

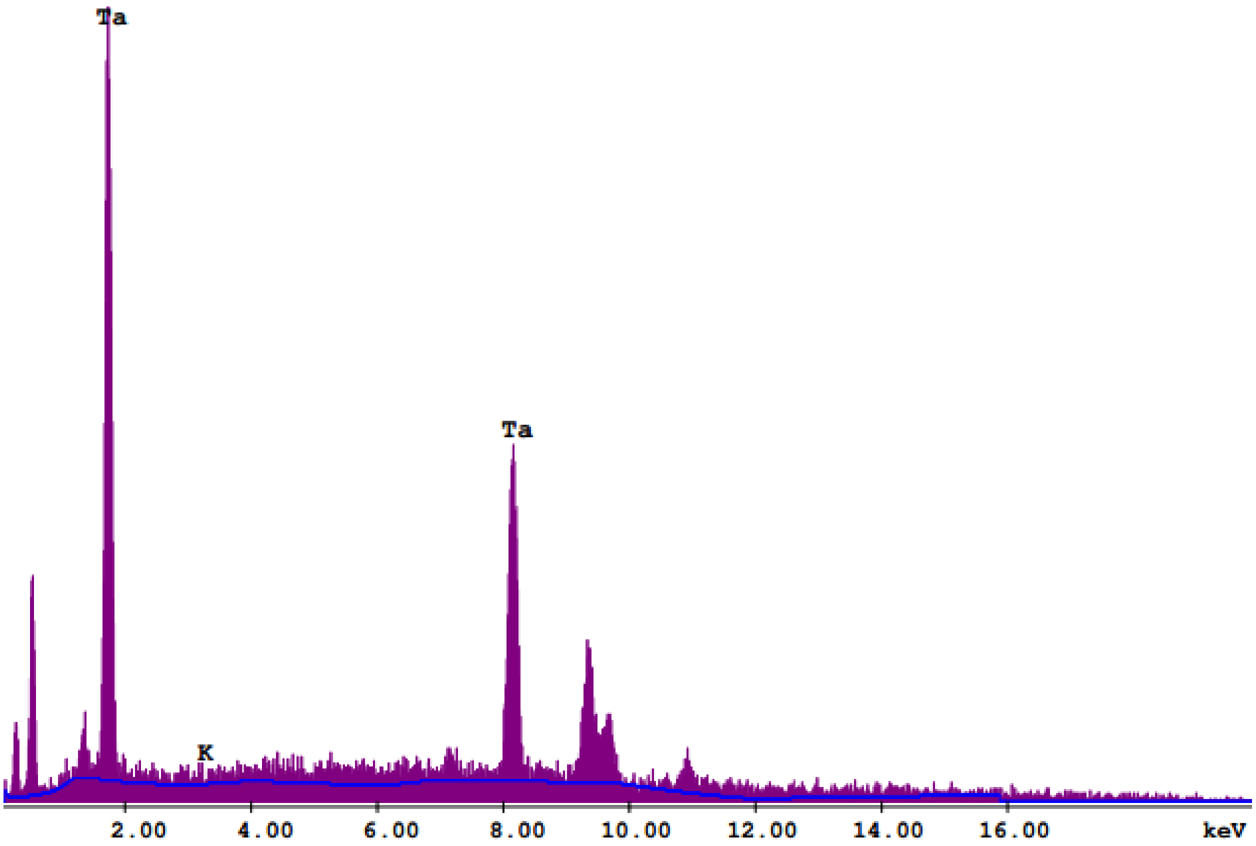

Figure S1: Sample EDX spectrum of $Li_8Ta_6O_{19}$, indicating the complete replacement of K^+ countercations (indistinguishable from background) by metathesis in 1M LiOH.

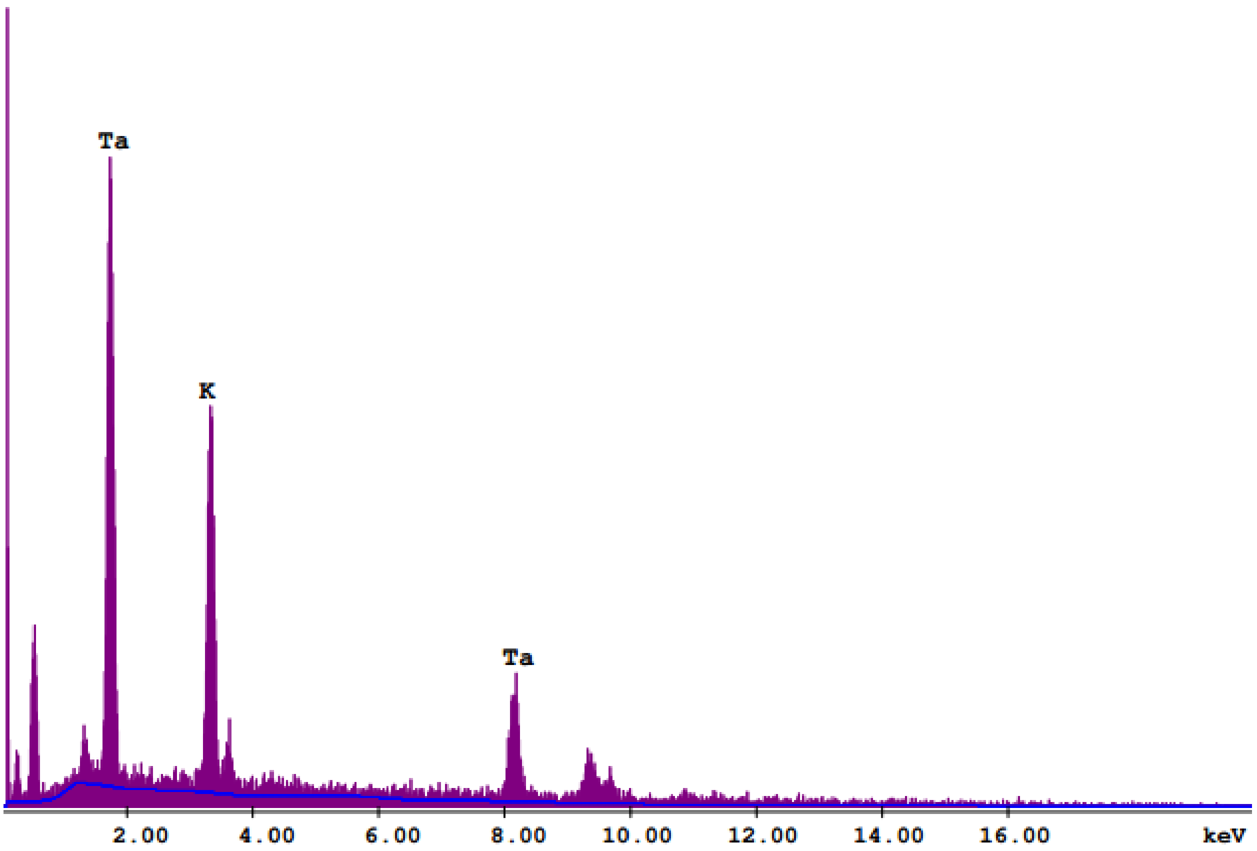

Figure S2: Sample EDX spectrum of $K_8Ta_6O_{19}$.

Table S14: Atom % values for K and Ta in $K_8Ta_6O_{19}$

Measurement #	at% K	at% Ta
1	59.97	40.03
2	55.59	44.41
3	60.14	39.86
4	52.85	47.15
5	58.72	41.28
Average	57.45	42.55
Expected	57.14	42.86

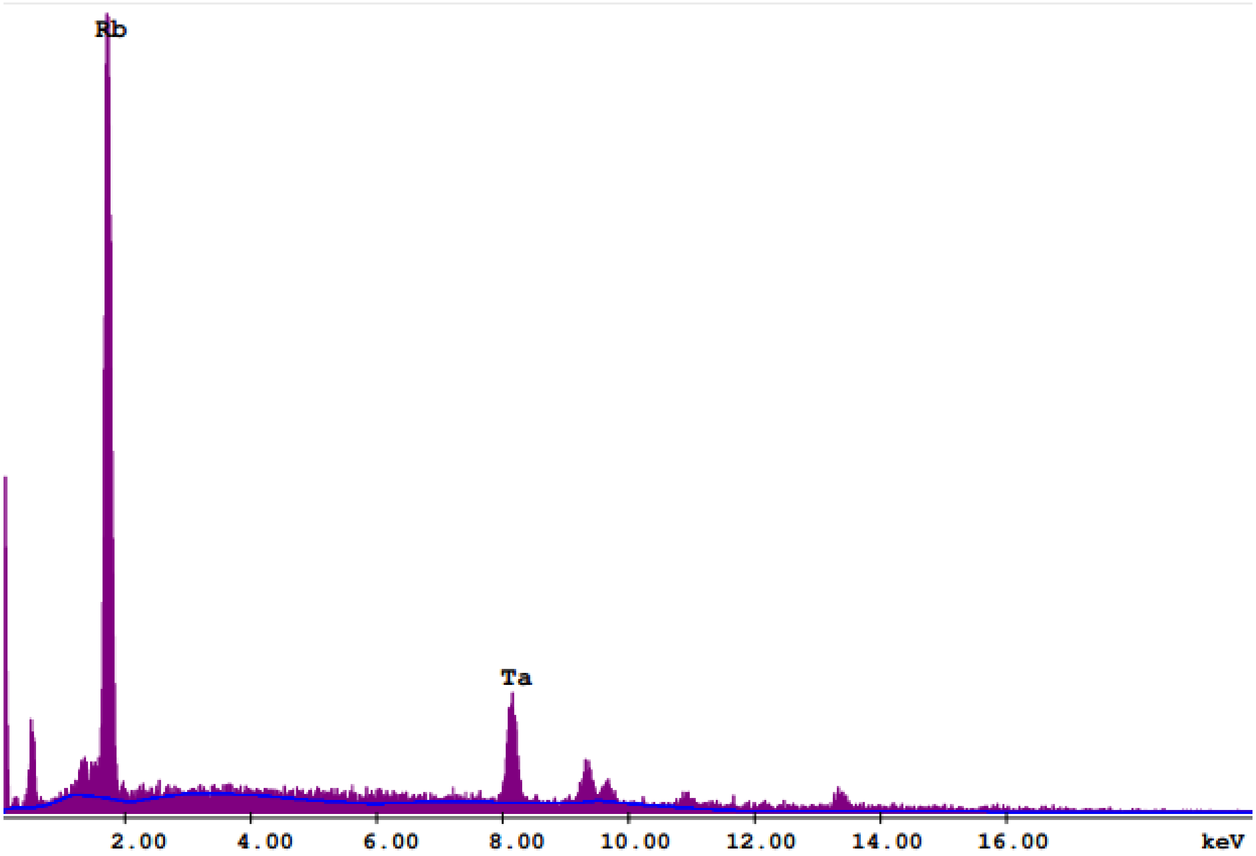

Figure S3: Sample EDX spectrum of $Rb_8Ta_6O_{19}$.

Table S15: Atom % values for Rb and Ta in $Rb_8Ta_6O_{19}$

Measurement #	at% Rb	at% Ta
1	58.42	41.58
2	56.38	43.62
3	56.82	43.18
4	57.93	42.07
5	56.39	43.61
Average	57.19	42.81
Expected	57.14	42.86

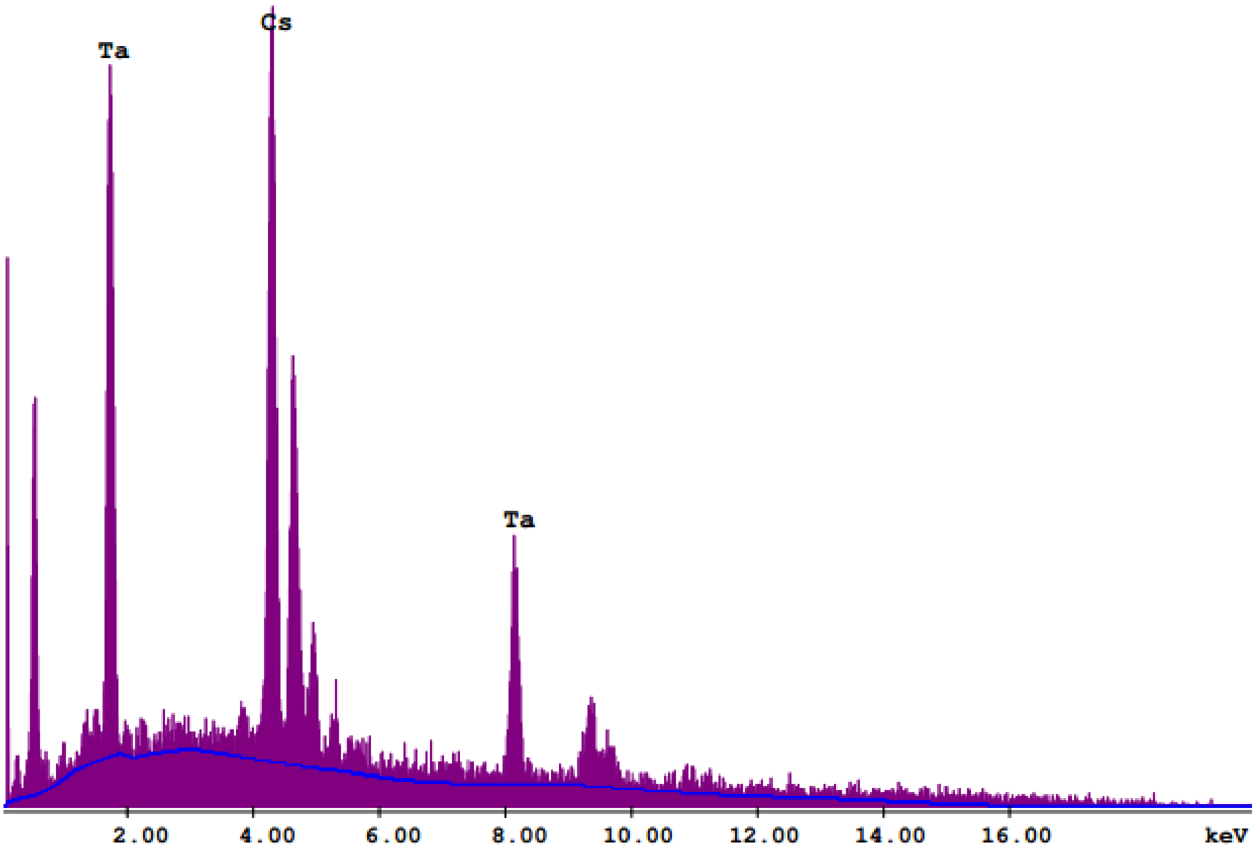

Figure S4: Sample EDX spectrum of $Cs_8Ta_6O_{19}$.

Table S16: Atom % values for Cs and Ta in $Cs_8Ta_6O_{19}$

Measurement #	at% Cs	at% Ta
1	58.56	41.44
2	56.82	43.18
3	59.72	40.28
4	59.65	40.35
5	59.71	40.29
Average	58.89	41.11
Expected	57.14	42.86

S4.2 Thermogravimetric Analysis

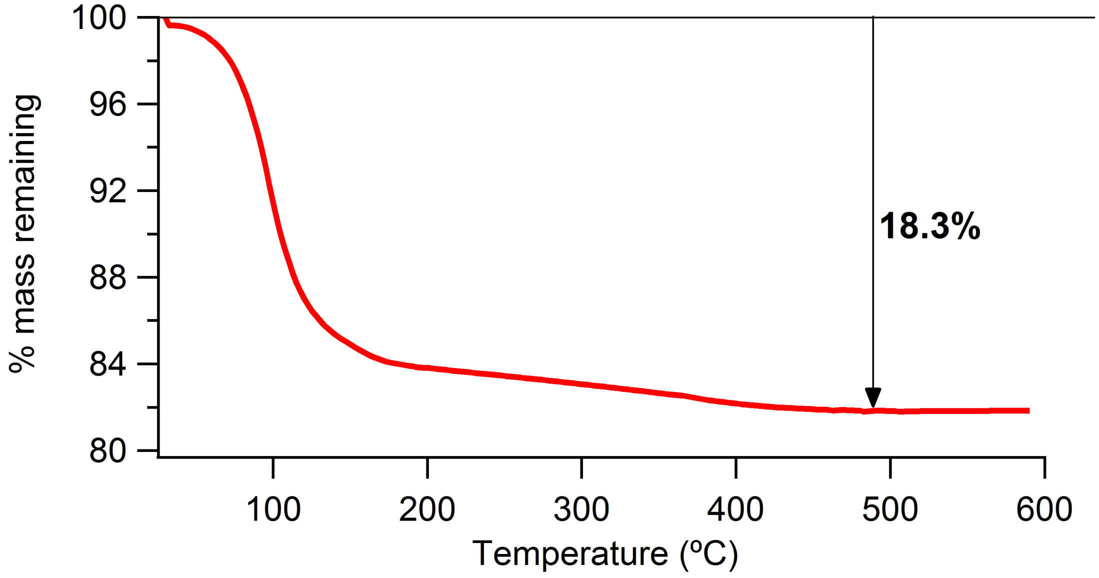

Figure S5: Thermogravimetric analysis of $Li_8Ta_6O_{19}$. All mass loss is due to lattice water, corresponding to 18 H_2O molecules per formula unit.

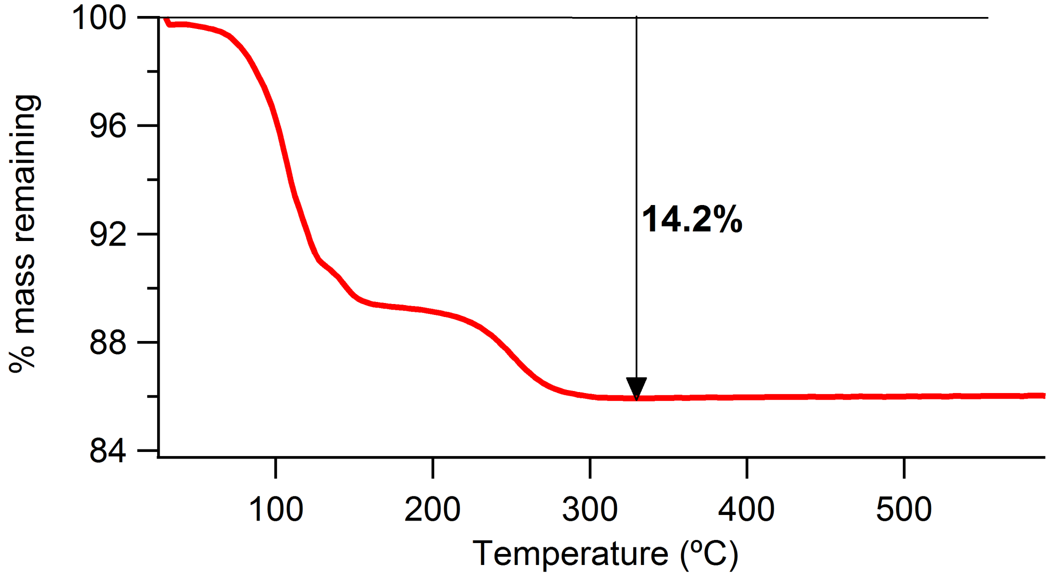

Figure S6: Thermogravimetric analysis of $K_8Ta_6O_{19}$. All mass loss is due to lattice water, corresponding to 16 H_2O molecules per formula unit.

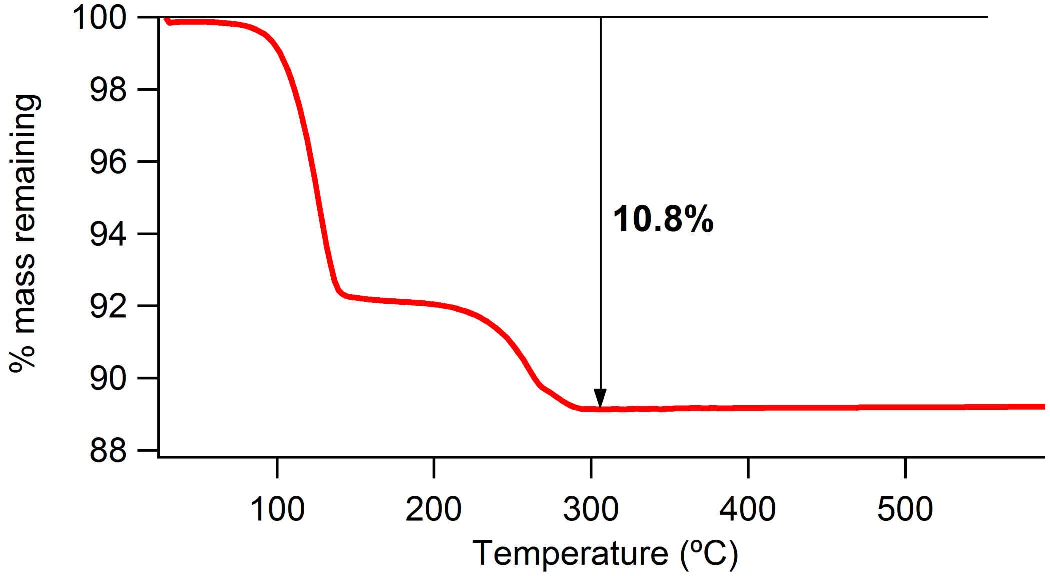

Figure S7: Thermogravimetric analysis of $Rb_8Ta_6O_{19}$. All mass loss is due to lattice water, corresponding to 14 H_2O molecules per formula unit.

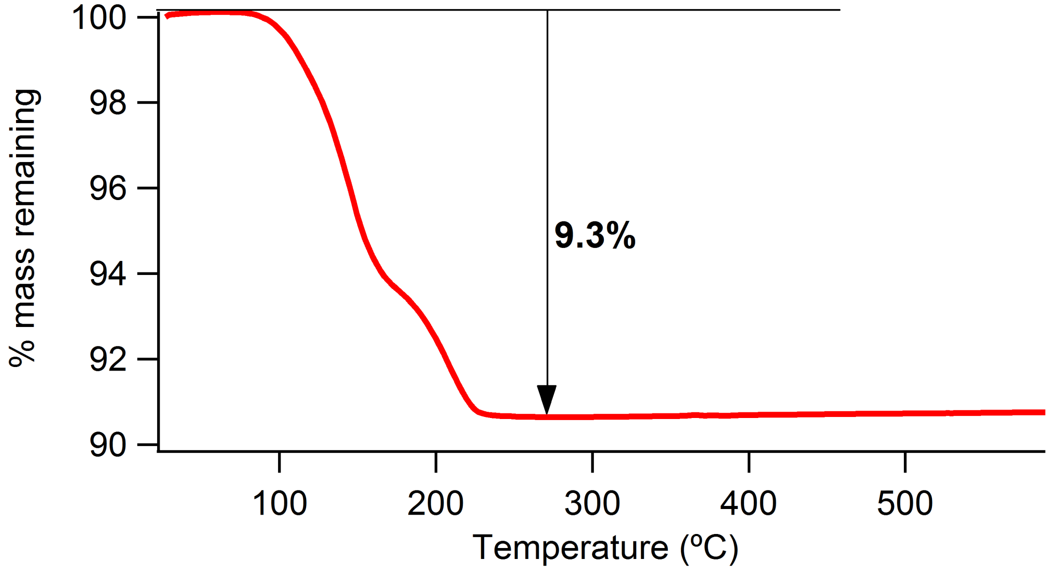

Figure S8: Thermogravimetric analysis of $Cs_8Ta_6O_{19}$. All mass loss is due to lattice water, corresponding to 14 H_2O molecules per formula unit.
